# *Neorickettsia helminthoeca* in Dog, Brazil

**DOI:** 10.3201/eid1208.060130

**Published:** 2006-08

**Authors:** Selwyn A. Headley, Diana G. Scorpio, Nicole C. Barat, Odilon Vidotto, J. Stephen Dumler

**Affiliations:** * Universidade Estadual de Londrina, Londrina, Brazil;; † Johns Hopkins University School of Medicine, Baltimore, Maryland, USA

**Keywords:** Neorickettsia helminthoeca, Anaplasmataceae, dogs, polymerase chain reaction, salmon poisoning disease, rrs, rpoB, groESL, letter

To the Editor: Neorickettsia helminthoeca causes salmon poisoning disease (SPD) in canids. SPD has been described only in the United States and the northwestern Pacific region of Canada (*1*). This report complements previous pathologic findings (*2*) and identifies SPD beyond the known disease-endemic region.

From 2001 to 2005, 20 dogs (5 mongrels and 15 beagles) showed pathologic lesions consistent with SPD. All beagles were born in coastal Florianópolis, Santa Catarina, Brazil, and later transferred to Maringá, Paraná, Brazil, for the last 3–4 years of life. Lymph nodes, spleen, liver, and intestines from 10 beagles were aseptically obtained at necropsy in Maringá and frozen at -20°C until used at the Johns Hopkins Medical Institutions in Baltimore, Maryland.

Genomic DNA was extracted from frozen tissues with QIAamp DNA Mini Kits (Qiagen, Valencia, CA, USA). DNA from N. helminthoeca and Anaplasma phagocytophilum was used as a positive control. Nuclease-free water was used as a negative control. We used gene-specific primers for Neorickettsia spp. 16S rRNA (rrs) (NeoSH-F; 5´-TAGGCCCGCGTTAGATTAGCTTGT-3´ and NeoSH-R; 5´-TACAACCCAAGGGCCTTCATCACT-3´) and N. helminthoeca RNA polymerase β-subunit (rpoB) (NH-rpoB-F: 5´-TGTCTTCGAAGGCCCAAAGACAGA-3´ and NH-rpoB-R: 5´-AGAACCGATAGAGCGGGCATGAAT-3´) (*3*) and heat-shock protein groESL (NH-groESL-F: 5´-AGGCTACTTCGCAGGCAAATGAGA-3´ and NH-groESL-R: 5´-CACGCTTCATTCCGCCCTTTAACT-3´) (*4**,**5*). Citrate synthase (gltA) gene primers (*6*) were also used. Two PCRs were conducted to maximize sensitivity.

Specificity of N. helminthoeca–specific primers was shown by amplification studies of genomic DNA of A. phagocytophilum, Ehrlichia chaffeensis, E. canis, N. risticii, N. sennetsu, and N. helminthoeca. All amplicons were separated by electrophoresis in 1% agarose gels and purified before cloning (pGEM-T and pGEM-T Easy Vector Systems, Promega, Madison, WI, USA) and sequencing. The Maringá sequences obtained were compared with those in GenBank by using BLAST (http://www.ncbi.nlm.nih.gov/BLAST). Phylogenetic trees, sequence alignments, and identity tables were created by using Vector NTI Advance10 Software (Invitrogen, Carlsbad, CA, USA). GenBank accession numbers of Anaplasmataceae and their phylogenetic relationships are shown in the Figure.

**Figure:**
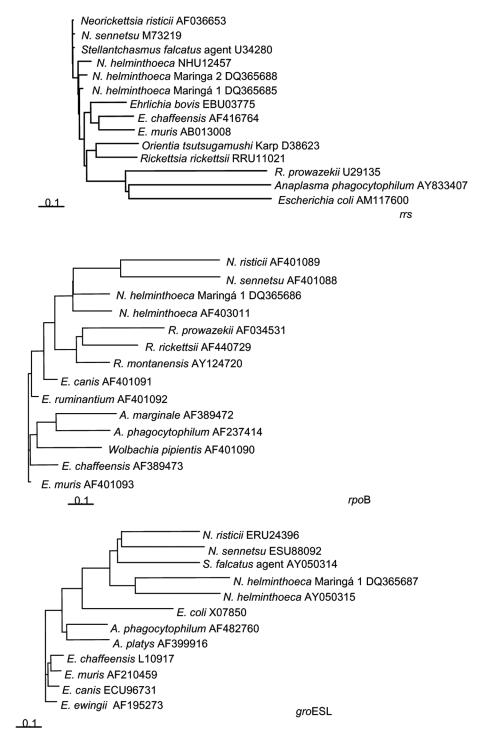
Neighbor-joining phylogenetic trees of the 16S rRNA (*rrs*), RNA polymerase β-subunit (*rpo*B), and heat-shock protein (*gro*ESL) gene sequences of *Anaplasmataceae* families. Trees were constructed with Vector NTI Advance10 Software (Invitrogen, Carlsbad, CA, USA). Bars represent substitutions per 1,000 bp. GenBank sequence accession numbers are provided.

Two dogs (N40–05, mesenteric lymph node, Maringá 1 and N20–04, Peyer's patch, Maringá 2) contained Neorickettsia spp. rrs, rpoB, or groESL genes. Both samples produced partial sequences for Neorickettsia spp. rrs gene; a similarity of 99% was observed for the 2 Maringá dog rrs sequences with N. sennetsu, N. risticii, and the Stellantchasmus falcatus (SF) agent. However, N. helminthoeca rpoB and groESL partial sequences were obtained only from dog 1. DNA identities of 100%, 82%, and 81% were observed between Maringá dog 1 sequences and N. helminthoeca, N. risticii, and N. sennetsu for the rpoB genes, respectively. All dogs were negative when tested with gltA gene primers. We observed 100% identity between the Maringá dog 1 sequence and N. helminthoeca groESL gene sequences. Similarities of 84%, 80%, and 79% were observed with N. sennetsu, the SF agent, and N. risticii, respectively. All positive controls showed bands of appropriate sizes, whereas negative controls yielded no products, confirming lack of amplicon contamination.

This study demonstrates that 2 dogs from Maringá, Brazil, with pathologic lesions consistent with SPD (*7*) were infected with a Neorickettsia sp. The partial sequences from dog 1 were identical to N. helminthoeca rrs, groESL, and rpoB genes, confirming infection with this organism (*2*). To our knowledge, this is the first confirmed description of this organism beyond the known geographic area of SPD. The organism identified in Brazil has been named N. helminthoeca Maringá strain.

Because of difficulty in recovering DNA from samples, need for a highly efficient PCR targeting small DNA regions, and limited sensitivity of the amplifications, sequences obtained for N. helminthoeca Maringá dog 1 (112 bp for rrs, 92 bp for groESL, 143 bp for rpoB) were short compared with those in GenBank (rrs 1,453 bp, groESL 1,914 bp, rpoB, 464 bp). Efficiency and sensitivity of targeting small DNA regions was necessary since storage and shipment of frozen samples were not optimal. Small DNA sequences are often suboptimal for delineation of phylogenetic relationships.

Bootstrapping analyses showed poor resolution (<380/1,000 iterations) below the genus level for the short rrs region examined. However, both the short rpoB and groESL regions examined had high bootstrap values (941/1,000 and 995/1,000 iterations, respectively). This finding allowed differentiation of N. helminthoeca and the Brazilian dog strain from N. sennetsu, N. risticii, and other related Anaplasmataceae and provided a high degree of confidence in the identification. More work is being implemented to obtain longer sequences to confirm and extend these genotypic comparisons. We propose further study to isolate the pathogen from other dogs for comparative biologic analyses.

Although SPD is caused by N. helminthoeca, infections by other Neorickettsia spp., including N. risticii (Potomac horse fever) and N. sennetsu (sennetsu fever), illustrate the potential of these widely distributed species to infect and cause disease in mammals and humans. Detection of N. helminthoeca in Brazilian dogs extends the range of this species and warrants a broad search for infections and spectrum of disease of Neorickettsia in animals and humans.
